# Different Brain Phenotypes in Magnetic Resonance Imaging of Healthy Children after Prenatal Insults

**DOI:** 10.3390/diagnostics12112748

**Published:** 2022-11-10

**Authors:** Cristina Paules, María Teresa Pérez Roche, Miguel Angel Marin, Nicolás Fayed, Gracián García-Martí, Javier López Pisón, Daniel Oros, Victoria Pueyo

**Affiliations:** 1 Obstetrics Department, Hospital Clínico Universitario Lozano Blesa, Avenida San Juan Bosco 15, 50009 Zaragoza, Spain; 2 Aragon Institute of Health Research (IIS Aragon), Avenida San Juan Bosco 13, 50009 Zaragoza, Spain; 3 Red de Salud Materno Infantil y del Desarrollo (SAMID), RETICS, Instituto de Salud Carlos III (ISCIII), Subdirección General de Evaluación y Fomento de la Investigación y Fondo Europeo de Desarrollo Regional (FEDER), Plaza de Cruces, 48903 Barakaldo, Spain; 4 Ophthalmology Department, Miguel Servet University Hospital, Paseo Isabel la Católica 1-3, 50009 Zaragoza, Spain; 5 Radiolology Department, Miguel Servet University Hospital, Paseo Isabel la Católica 1-3, 50009 Zaragoza, Spain; 6 Radiology Department, Quirónsalud Hospital, Mariano Renovales, 50006 Zaragoza, Spain; 7 Unidad de Ingeniería Biomédica, Hospital Quirónsalud, Blasco Ibáñez 14, 46010 Valencia, Spain; 8 CIBERSAM, Institute of Health Carlos III, Monforte de Lemos 3-5, 28029 Madrid, Spain; 9 Neuropediatrics Department, Miguel Servet University Hospital, Paseo Isabel la Católica 1-3, 50009 Zaragoza, Spain

**Keywords:** preterm birth, small for gestational age, maternal behavior/drug effects, magnetic resonance imaging

## Abstract

In this study, we used magnetic resonance imaging (MRI) to identify the different brain phenotypes within apparently healthy children and to evaluate whether these phenotypes had different prenatal characteristics. We included 65 healthy children (mean age, 10 years old) with normal neurological examinations and without structural abnormalities. We performed cluster analyses to identify the different brain phenotypes in the brain MRI images. We performed descriptive analyses, including demographic and perinatal characteristics, to assess the differences between the clusters. We identified two clusters: Cluster 1, or the “small brain phenotype” (n = 44), which was characterized by a global reduction in the brain volumes, with smaller total intracranial volumes (1044.53 ± 68.37 vs. 1200.87 ± 65.92 cm^3^ (*p* < 0.001)), total grey-matter volumes (644.65 ± 38.85 vs. 746.79 ± 39.37 cm^3^ (*p* < 0.001)), and total white-matter volumes (383.68 ± 40.17 vs. 443.55 ± 36.27 cm^3^ (*p* < 0.001)), compared with Cluster 2, or the “normal brain phenotype” (n = 21). Moreover, almost all the brain areas had decreased volumes, except for the ventricles, caudate nuclei, and pallidum areas. The risk of belonging to “the small phenotype” was 82% if the child was preterm, 76% if he/she was born small for his/her gestational age and up to 80% if the mother smoked during the pregnancy. However, preterm birth appears to be the only substantially significant risk factor associated with decreased brain volumes.

## 1. Introduction

Fetal programming is defined as the environment in the uterus during specific critical periods of the pregnancy. Its disturbance can lead to long-term alterations in the development of the fetus and child [1,2]. David Barker developed the concept of critical windows during fetal development, which are sensitive to different nutritional, hormonal and metabolic insults [3]. The development of the central nervous system starts in the third week of gestation and progresses throughout pregnancy [4]. Thus, the nervous system has an important susceptibility to prenatal insults, and these brain-development disturbances can extend into adulthood [5,6]. 

A growing body of evidence supports the lasting adverse effect of prenatal exposure to maternal cigarette use [7,8,9], which is associated with substantial reductions in the cortical gray matter, total parenchymal volumes, and head circumference measured by MRI at school age [10]. Moreover, being born small for gestational age (SGA) and/or preterm have been described as risk factors for abnormal brain development that can lead to neurological and intellectual dysfunctions [11,12,13,14]. In most studies performed during childhood, researchers describe neurostructural changes that are associated with neurological impairment in severe cases of SGA or preterm children [15,16,17]. However, the impacts of these prenatal insults on the brain structures of apparently healthy children are still poorly defined.

In this study, our main objective was to explore the existence of the heterogeneity expressed by different brain MRI phenotypes within apparently healthy children, and to evaluate whether these phenotypes have different prenatal characteristics. To this aim, we performed MRI examinations on the brains of 65 healthy children, and we performed a cluster analysis to identify the structural brain phenotypes among these children. Then, we compared the prenatal characteristics among the different clusters, and we evaluated the risk of belonging to each cluster. According to our results, premature birth was the only risk factor for decreased brain volumes among those evaluated. 

## 2. Materials and Methods

The study protocol was approved by the local ethics committee (Comité de Ética de la Investigación de la Comunidad de Aragón (CEICA)) (expedient number: CI PI12/0021; date of approval: 12 March 2012). The president of the CEICA was César Loris. We obtained written informed consent from the parents or guardians of each child. We blinded the examiners to the perinatal data and study groups. All the procedures adhered to the tenets of the Declaration of Helsinki.

### 2.1. Participants

We performed brain MRI examinations on the children in a clinical setting at the presence of minor neurological symptoms (mostly headache or dizziness) between January 2012 and April 2016 in a tertiary university hospital. We excluded subjects with confirmed or suspected genetic defects, congenital malformations and abnormalities in neonatal brain ultrasonographies, or any cognitive or motor impairment, from the study. We also excluded children with previous pediatric events causing neurological diseases, such as meningitis/sepsis, craniocerebral trauma or cardiorespiratory events requiring ICU care. In addition, a pediatric neurologist assessed all the children for inclusion in the study. We only invited children with normal neurological examinations to participate. 

We collected the demographic and pediatric information from the medical records of the hospital, including the gestational age at birth, birthweight, sex, obstetric characteristics, maternal smoking during pregnancy, perinatal outcomes, and childhood diseases. We defined the SGA cohort as birthweights below the 10th centile, according to local standards [18]. We defined the fetal growth restriction by birthweights below the 3rd centile [19].

We confirmed the gestational age by the length of the first trimester crown rump [20]. We considered those children born before 37 weeks as preterm.

### 2.2. MRI Data Acquisition and Analysis

We used the 1.5-T Optima 360 Advance clinical scanner (GE Healthcare Diagnostic Imaging, Milwaukee, WI, USA) to obtain the three-dimensional (3D) gradient-echo T1-weighted whole-brain images using a 16-channel array head coil. We performed a 3D high-resolution structural MRI (1 mm isotropic voxels) examination on every child using a T1-weighted volumetric spoiled gradient-recall echo sequence (repetition time: 9.1 ms; echo time: 1.7 ms; flip angle: 20°; number of excitations: 1; matrix size: 256Å~256; slice thickness: 1.5 mm; gap: 0; yield: 124 transverse slices; voxel size: 0.86Å~0.86Å~1.5 mm). A neuroradiologist and computer engineer, both of whom were blinded to the children’s clinical information, reviewed all the images. We excluded participants from the study if they found structural brain abnormalities upon this first inspection. There were no exclusions at this step. Once anonymized, we transferred the images for analysis and postprocessing to a workstation. We used two spatial filters for increasing the signal-to-noise ratio and for the semiautomatic quantitative volumetry. The first process consisted of a nonlocal spatial filter that we used to decrease the fluctuation in the MR signal due to the thermal noise. After that, we also corrected the filtered images for MR inhomogeneities. We assessed the volumes of the different brain areas by parcellation with the FreeSurfer software package (http://surfer.nmr.mgh.harvard.edu/ (accessed on 1 September 2016)). With this method, we were able to the extract the white- and grey-matter surfaces and assess the volumes of the cortical and subcortical areas. We measured the total grey matter and total white matter, as well as the volumes of the following: the lateral ventricles; frontal lobe; parietal lobe; temporal lobe; occipital lobe; cingulum; thalamus; putamen; caudate nucleus; globus pallidus; hippocampus; cerebellum (grey and white matter); and amygdala. Two trained engineers evaluated the anatomical accuracies of the grey- and white-matter parcellations.

### 2.3. Statistical Analyses

We performed the statistical analyses using SPSS V.21.0 (SPSS, Chicago, IL, USA) statistical software. We analyzed all the MRI examinations by cluster analysis, which is an exploratory and data-mining technique for identifying heterogeneity [21,22]. We created several groups according to the similarities of the individuals so that the children in the same group were similar to each other but different from those in the other groups. We present the hierarchical cluster analysis and possible subgroups in a dendrogram graph. We used the nonhierarchical K-means method to confirm the number of clusters and create the subgroups. Then, we used descriptive analysis, taking into account the demographic and perinatal characteristics, to define the clusters and assess the differences between them. We applied the Kolmogorov–Smirnov test to assess the sample distributions for all the quantitative outcomes. We performed a comparison of the study groups with the Student’s *t*-test, Mann–Whitney U test, or χ^2^ test as appropriate. We performed the Bonferroni correction for multiple comparisons. We calculated the odds ratio (OR) with a 95% confidence interval by logistic regression.

## 3. Results

### 3.1. Brain MRI Phenotypes 

**Figure 1:**
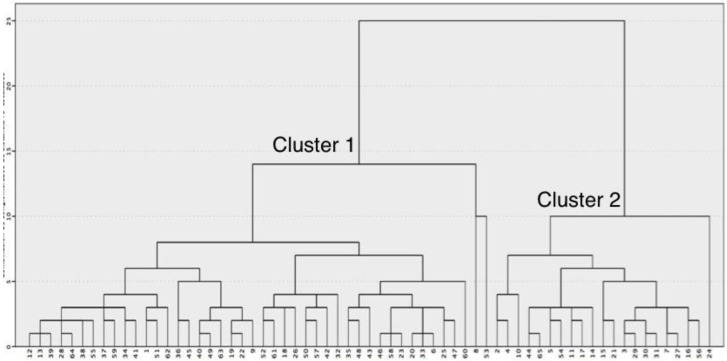
Dendrogram obtained from hierarchical cluster analysis of MRI brain volumes and dissimilarity measures between groups. Cluster analysis identified heterogeneity by grouping patients so that the individuals in the same group were similar to each other but different from those in the other groups.

By using cluster analysis with the hierarchical approach and Wards linkage, we identified two different groups among the 65 brain MRI examinations performed (Figure 1). According to the K-means, two clusters could better represent the differences among the subgroups. 

We present the differences in the brain volumes of the two identified phenotypes in Table 1. Cluster 1, or the “small brain phenotype” (n = 44), was characterized by a global reduction in the brain volumes, with smaller total intracranial volumes (1044.53 ± 68.37 vs. 1200.87 ± 65.92 cm^3^ (*p* < 0.001)), total grey-matter volumes (644.65 ± 38.85 vs. 746.79 ± 39.37 cm^3^ (*p* < 0.001)), and total white-matter volumes (383.68 ± 40.17 vs. 443.55 ± 36.27 cm^3^ (*p* < 0.001)) compared with Cluster 2, or the “normal brain phenotype” (n = 21). Moreover, almost all the brain areas had decreased brain volumes, except for the ventricles, caudate nuclei, and pallidum areas. Interestingly, the lateral (right: 3.02 (3.37) vs. 2.87 (2.87) cm^3^ (*p* = 0.501); left: 3.80 (4.31) vs. 4.04 (3.04) cm^3^ (*p* = 0.319)), third (0.75 (0.28) vs. 0.68 (0.38) cm^3^ (*p* = 0.123)) and fourth (1.58 ± 0.49 vs. 1.58 ± 0.71 cm^3^ (*p* = 1)) ventricles presented similar results between groups, which were not discriminatory. 

**Figure 2:**
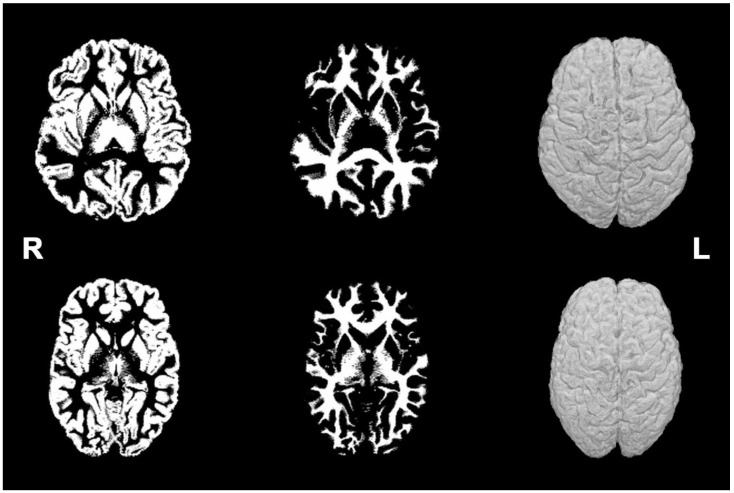
Representative MRI brain images for two phenotypes (gray matter, white matter, and rendered 3D surface). (**Top row**): normal subject. (**Bottom row**): Cluster 1 or “small brain phenotype” subject.

We present a comparison of the brain MRI images between both clusters, which shows the global reduction in the brain volumes in those patients included in Cluster 1, in Figure 2. 

### 3.2. Perinatal Characteristics of Different Brain MRI Phenotypes 

We present the characteristics of the two brain MRI clusters that we obtained in Table 2. Both groups had similar results regarding age and sex. According to our data, the children with the “small brain phenotype” were more frequently born preterm (52.3% of the premature children in Cluster 1 vs. 23.8% of the premature children in Cluster 2), had lower birthweights (2240 ± 975 vs. 3053 ± 924), and included the highest prevalence of patients with two or more of the subsequent risk factors: prematurity, SGA, and maternal smoking (40.9% vs. 14.3%). 

**Figure 3:**
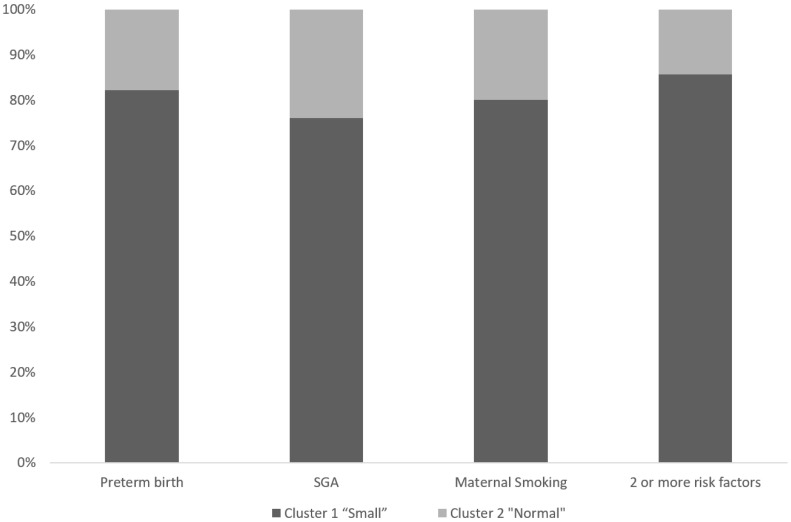
Probability of belonging to each phenotype according to perinatal characteristics.

Finally, we analyzed the probability of belonging to the “small brain phenotype” according to the perinatal characteristics. We reported a risk of 82% if the child was preterm, 76% if the child was born SGA, and 80% if the mother smoked during the pregnancy. We also observed that those children with two or more of the subsequent risk factors (prematurity, SGA, and maternal smoking) had an 85% risk of suffering a global reduction in their brain volumes (Figure 3). Premature birth had the highest impact of all these factors (OR: 3.5 (1.1–11.2)).

## 4. Discussion

In this study, we describe the presence of two different brain MRI phenotypes (small and normal) in healthy children with normal neurodevelopment and neurological examinations, and without structural abnormalities. The risk of belonging to the “small brain phenotype”, which is characterized by a global reduction in the brain volume, seems to mainly be influenced by preterm birth. Other factors, such as maternal smoking or being SGA, did not reach statistical significance in our sample, despite the higher frequency in the group of children with smaller brain volumes. 

However, our data support the impact of certain prenatal insults on the brain structure during childhood, which supports the concept of “fetal programming”. We hypothesized that these prenatal insults might not be so severe as to induce important neurodevelopmental consequences, but severe enough to produce structural changes in the brains of these children. Thus, despite being apparently healthy children, they presented certain degrees of brain remodeling. 

Brain development is a complex process that involves the maturation and functional specialization of the grey-matter regions and the establishment and myelination of the white-matter connections between the brain regions. Life-long developmental neuroplasticity is formed during critical periods of brain maturation over these first 1000 days [23,24]. Fetal growth restriction and prematurity are adverse outcomes that are often based on ischemic placental disease from defective trophoblastic precursor development and inadequate vascular development, with the suboptimal first-trimester placental development of the blastocyst shortly after conception [25]. Then, the trimester-specific processes that affect the maternal–placental–fetal triad might play an important role in conferring the changes in the brain structure that persist into childhood, even in apparently healthy children [26]. 

Our findings are consistent with previous studies, in which the authors report specific neurostructural anomalies in SGA and preterm children [27,28]. Researchers have associated fetal smallness with adult-related neurologic problems and alterations in the brain tissue volume and function [29]. They also report that premature children have reduced total brain volumes, smaller cortical surface areas and lower volumes of grey matter compared with controls [30,31]. In our sample, prematurity was more associated with a reduction in the brain volume than SGA, which might be because prematurity was a pathological insult in all the cases, whereas in some of the cases, SGA was a constitutional issue rather than a pathological one. Moreover, in most studies, researchers report a reduced ponderal index, which is a measure of the fetal growth restriction and is more pervasive than IUGR (<3%) and SGA (<10%). Moreover, in previous studies, researchers observed an inverse relationship between prenatal cigarette exposure and general intelligence, which persisted into adolescence [8]. Researchers have also associated this exposure with substantial reductions in the cortical gray matter, total parenchymal volumes, and head circumferences of school-aged children measured by MRI [10]. However, with our study, we introduce a novel approach to identifying heterogeneity in the brain structure during childhood and to understanding why some of these children are triggered to develop the phenotype of brain reduction, compared to thise who do not. 

Contrary to our expectations, the enlargement of the ventricles was not a discriminatory marker, as both phenotypes presented similar ventricular sizes. Preterm babies usually present enlarged ventricles compared with controls [32,33]. However, in most studies, researchers include severe cases of preterm children with different brain lesions such as interventricular hemorrhages and periventricular leukomalacia [34]. The enlargement of the ventricles might then be associated with moderate–severe cases or related to these brain lesions. Because we detected subtle quantitative variations in the brains of children during childhood in our study, the ventricle changes were not so clear in our cohort. 

The high prevalence of these prenatal insults (maternal smoking, prematurity, and SGA) makes this population a substantial public health concern. The prenatal care and counsel of pregnant women should include an emphasis on the potential lasting consequences of the prenatal exposure to cigarettes on the brain structure, and assistance should be offered to reduce the use of this substance. Future research is needed to clarify the underlying mechanisms that are responsible for this brain remodeling. Moreover, these brain changes could be associated with subtle neurodevelopmental impairments that have not been detected because these children are considered healthy. A fetal/neonatal program that applies this perspective will better identify the trimester-specific mechanisms that affect the maternal–placental–fetal triad, which are expressed as brain changes. Finally, the early identification of this high-risk group might be an open opportunity for more suitable follow-up and timely postnatal interventional strategies, which could improve the short- and long-term outcomes of these children. 

This study has some strengths and limitations. To the best of our knowledge, this is the first attempt to explore different brain phenotypes related to prenatal insults in apparently healthy children by using cluster analysis, which is a novel approach in fetal medicine but is already used in other research areas. Cluster analysis has advantages over the pure description of the means/medians by allowing for the identification of the clinical heterogeneity and an understanding of why some patients might be predisposed to disease whereas others (with similar risk factors) are not. However, this is a noninferential technique that needs to be validated in other populations. Another limitation is the small sample size, which impeded the obtainment of statistically significant differences in some outcomes with prominent tendencies. Our study had restrictive inclusion criteria, and we only included healthy children in our sample size. We excluded any children with neurologic impairments or abnormal findings in their brain structures detected either by neonatal ultrasonography or brain MRI, which made it difficult to increase the number of included children. Despite the exclusion of subjects with confirmed or suspected genetic defects, we did not perform microarray, exomic or genomic sequencing for the inclusion or exclusion of the children. This examination did not allow us to detect substantial neurological impairments, but researchers could use specific tests to reveal more subtle neurodevelopmental deficits. We encourage the further assessment of our findings with larger samples and more extensive testing.

## Figures and Tables

**Table 1:** Differences in brain volumes (cm^3^) between two phenotypes of brain MRI examinations identified by cluster analysis.

	Cluster 1 “Small” (N = 44)	Cluster 2 “Normal” (N = 21)	*p*-Value
Total intracranial volume	1044.53 ± 68.37	1200.87 ± 65.92	<0.001
Total grey matter	644.65 ± 38.85	746.79 ± 39.37	<0.001
Total white matter	383.68 ± 40.17	443.55 ± 36.27	<0.001
Lateral ventricle (right)	3.02 (3.37)	2.87 (2.87)	0.501
Lateral ventricle (left)	3.80 (4.31)	4.04 (3.04)	0.319
Third ventricle	0.75 (0.28)	0.68 (0.38)	0.123
Fourth ventricle	1.58 ± 0.49	1.58 ± 0.71	1
Frontal lobe (right)	84.0 ± 7.58	99.02 ± 6.39	<0.001
Frontal lobe (left)	83.77 ± 7.66	98.62 ±7.63	<0.001
Temporal lobe (right)	50.89 ± 5.60	59.92 ± 6.25	<0.001
Temporal lobe (left)	53.0 ± 6.04	62.19 ± 6.17	<0.001
Parietal lobe (right)	59.79 ± 4.35	70.52 ± 5.19	<0.001
Parietal lobe (left)	57.41 ± 4.67	67.64 ± 5.06	<0.001
Occipital lobe (right)	24.27 ±3.15	27.37 ± 3.06	<0.001
Occipital lobe (left)	23.74 ± 2.64	27.35 ± 3.21	<0.001
Cingulum (right)	9.18 ±1.24	10.71 ± 1.43	<0.001
Cingulum (left)	9.45 ± 1.16	11.34 ±1.87	<0.001
Thalamus (right)	6.89 ± 0.71	7.58 ± 0.49	<0.001
Thalamus (left)	7.13 ± 0.71	8.30 ± 0.83	<0.001
Caudate nucleus (right)	3.33 ± 0.48	3.72 ± 0.51	0.004
Caudate nucleus (left)	3.36 ± 0.45	3.76 ± 0.48	0.002
Putamen (right)	5.10 ± 0.59	5.71 ± 0.54	<0.001
Putamen (left)	5.17 ± 0.56	5.89 ± 0.65	<0.001
Pallidum (right)	1.44 ± 0.23	1.54 ± 0.27	0.148
Pallidum (left)	1.62 ± 0.28	1.72 ± 0.23	0.162
Hippocampus (right)	3.97 ± 0.40	4.52 ± 0.43	<0.001
Hippocampus (left)	3.95 ± 0.50	4.59 ± 0.43	<0.001
Amygdala (right)	1.34 ± 0.16	1.58 ± 0.23	<0.001
Amygdala (left)	1.34 ±0.15	1.55 ± 0.17	<0.001
Cerebellum grey matter (right)	55.87 ± 5.78	62.46 ± 6.11	<0.001
Cerebellum grey matter (left)	54.57 ± 5.69	60.81 ± 4.97	<0.001
Cerebellum white matter (right)	11.73 ± 1.83	13.51 ± 1.73	<0.001
Cerebellum white matter (left)	11.60 ± 1.53	133.21 ± 1.51	<0.001

Data are means ± standard deviations or medians (interquartile ranks). We calculated the *p*-values by Student’s *t*-tests or Mann–Whitney U tests, as appropriate. We applied a Bonferroni correction coefficient of 33 (number of included cerebral measurements) to a *p*-value of 0.05. Therefore, we only considered *p*-values < 0.001 as significant.

**Table 2:** Demographic and perinatal characteristics of two different brain MRI phenotypes.

	Cluster 1 “Small” (N = 44)	Cluster 2 “Normal” (N = 21)	*p*-Value
Age (years)	10.5 ± 2.7 (6.1–14.1)	10.4 ± 2.1 (6.5–14.6)	0.89
Male gender (%)	45.5	66.7	0.186
Maternal smoking (%)	27.3	14.3	0.35
Maternal education (%)			0.815
No studies	9.1%	7.1%	
Medium	42.4%	28.6%	
High education	42.4%	64.3%	
Gestational age at delivery (weeks)	35.5 (9)	40 (5)	0.003
Birthweight (gr)	2240 ± 975	3053 ± 924	0.002
Birthweight percentile	14 (65)	50 (83)	0.079
Preterm birth (%)	52.3	23.8	0.036
Severe preterm birth (%)	29.5	9.5	0.12
SGA (%)	43.2	28.6	0.29
Fetal growth restriction (%)	25	14.3	0.52
2 or more risk factors *	40.9	14.3	0.047

Data are percentages (%), means ± standard deviations or medians (interquartile ranks). We calculated the *p*-values by Student’s t-tests, Mann–Whitney U tests, or Pearson or χ^2^ tests, as appropriate. * Risk factors (preterm birth, SGA, and maternal smoking). SGA: small for gestational age; severe preterm birth (birth before 28 weeks of gestation); and fetal growth restriction (birthweight less than third percentile).

## Data Availability

The data associated with the paper are not publicly available, but they are available from the corresponding author upon reasonable request.

## References

[1] Barker D.J., Osmond C. (1986). Infant mortality, childhood nutrition, and ischaemic heart disease in England and Wales. Lancet.

[2] Barker D. (1990). The fetal and infant origins of adult disease. BMJ Br. Med. J..

[3] Barker D.J., Godfrey K., Gluckman P., Harding J., Owens J., Robinson J. (1993). Fetal nutrition and cardiovascular disease in adult life. Lancet.

[4] Volpe J.J. (2000). Overview: Normal and abnormal human brain development. Ment. Retard. Dev. Disabil. Res. Rev..

[5] Lister J.P., Blatt G.J., DeBassio W.A., Kemper T.L., Tonkiss J., Galler J.R., Rosene D.L. (2005). Effect of prenatal protein malnutrition on numbers of neurons in the principal cell layers of the adult rat hippocampal formation. Hippocampus.

[6] Rehn A., Buuse M.V.D., Copolov D., Briscoe T., Lambert G., Rees S. (2004). An animal model of chronic placental insufficiency: Relevance to neurodevelopmental disorders including schizophrenia. Neuroscience.

[7] Levin E.D., Wilkerson A., Jones J.P., Christopher N.C., Briggs S.J. (1996). Prenatal nicotine effects on memory in rats: Pharmacological and behavioral challenges. Dev. Brain Res..

[8] Fried P., Watkinson B. (2001). Differential effects on facets of attention in adolescents prenatally exposed to cigarettes and marihuana. Neurotoxicology Teratol..

[9] Slikker W., Xu Z.A., Slotkin T.A. (2005). Mode of action: Disruption of brain cell replication, second messenger, and neurotransmitter systems during development leading to cognitive dysfunction developmental neurotoxicity of nicotine. Crit. Rev. Toxicol..

[10] Rivkin M.J., Davis P.E., Lemaster J.L., Cabral H.J., Warfield S.K., Mulkern R.V., Robson C.D., Rose-Jacobs R., Frank D.A. (2008). Volumetric MRI Study of Brain in Children With Intrauterine Exposure to Cocaine, Alcohol, Tobacco, and Marijuana. Pediatrics.

[11] Colella M., Frérot A., Novais A.R.B., Baud O. (2018). Neonatal and Long-Term Consequences of Fetal Growth Restriction. Curr. Pediatr. Rev..

[12] Luu T.M., Rehman Mian M.O., Nuyt A.M. (2017). Long-Term Impact of Preterm Birth: Neurodevelopmental and Physical Health Outcomes. Clin. Perinatol..

[13] De Bie H.M.A., Oostrom K.J., Delemarre-van de Waal H.A. (2010). Brain development, intelligence and cognitive outcome in children born small for gestational age. Horm. Res. Paediatr..

[14] Nosarti C., Nam K.W., Walshe M., Murray R.M., Cuddy M., Rifkin L., Allin M.P. (2014). Preterm birth and structural brain alterations in early adulthood. Neuroimage Clin..

[15] Miller S.L., Hüppi P., Mallard C. (2016). The consequences of fetal growth restriction on brain structure and neurodevelopmental outcome. J. Physiol..

[16] Mathur A.M., Neil J.J., Inder T.E. (2010). Understanding Brain Injury and Neurodevelopmental Disabilities in the Preterm Infant: The Evolving Role of Advanced Magnetic Resonance Imaging. Semin. Perinatol..

[17] Nam K.W., Castellanos N., Simmons A., Froudist-Walsh S., Allin M.P., Walshe M., Murray R.M., Evans A., Muehlboeck J.-S., Nosarti C. (2015). Alterations in cortical thickness development in preterm-born individuals: Implications for high-order cognitive functions. NeuroImage.

[18] Figueras F., Meler E., Iraola A., Eixarch E., Coll O., Francis A., Gratacos E., Gardosi J. (2008). Customized birthweight standards for a Spanish population. Eur. J. Obstet. Gynecol. Reprod. Biol..

[19] Gordijn S.J., Beune I.M., Thilaganathan B., Papageorghiou A., Baschat A.A., Baker P.N., Silver R.M., Wynia K., Ganzevoort W. (2016). Consensus definition of fetal growth restriction: A Delphi procedure. Ultrasound Obstet. Gynecol..

[20] Robinson H.P., Fleming J.E.E. (1975). A Critical Evaluation of Sonar “Crown-Rump Length” Measurements. BJOG Int. J. Obstet. Gynaecol..

[21] Tzeng C.-R., Chang Y.-C.I., Chang Y.-C., Wang C.-W., Chen C.-H., Hsu M.-I. (2014). Cluster analysis of cardiovascular and metabolic risk factors in women of reproductive age. Fertil. Steril..

[22] Rodríguez-López M., Cruz-Lemini M., Valenzuela-Alcaraz B., Garcia-Otero L., Sitges M., Bijnens B., Gratacós E., Crispi F. (2017). Descriptive analysis of different phenotypes of cardiac remodeling in fetal growth restriction. Ultrasound Obstet. Gynecol..

[23] Scher M.S. (2021). “The First Thousand Days” Define a Fetal/Neonatal Neurology Program. Front. Pediatr..

[24] Scher M.S. (2022). Gene-Environment Interactions During the First Thousand Days Influence Childhood Neurological Diagnosis. Semin. Pediatr. Neurol..

[25] Brosens I., Puttemans P., Benagiano G. (2019). Placental bed research: I. The placental bed: From spiral arteries remodeling to the great obstetrical syndromes. Am. J. Obstet. Gynecol..

[26] Boyce W.T., Sokolowski M.B., Robinson G.E. (2020). Genes and environments, development and time. Proc. Natl. Acad. Sci. USA.

[27] Soria-Pastor S., Padilla N., Zubiaurre-Elorza L., Ibarretxe-Bilbao N., Botet F., Costas-Moragas C., Falcon C., Bargallo N., Mercader J.M., Junqué C. (2009). Decreased Regional Brain Volume and Cognitive Impairment in Preterm Children at Low Risk. Pediatrics.

[28] Arhan E., Gücüyener K., Soysal Ş., Şalvarlı Ş., Gürses M.A., Serdaroğlu A., Demir E., Ergenekon E., Türkyılmaz C., Önal E. (2017). Regional brain volume reduction and cognitive outcomes in preterm children at low risk at 9 years of age. Childs Nerv. Syst..

[29] Muller M., Sigurdsson S., Kjartansson O., Jonsson P.V., Garcia M., von Bonsdorff M.B., Gunnarsdottir I., Thorsdottir I., Harris T.B., van Buchem M. (2014). Birth Size and Brain Function 75 Years Later. Pediatrics.

[30] Inder T.E., Warfield S.K., Wang H., Hüppi P.S., Volpe J.J. (2005). Abnormal Cerebral Structure Is Present at Term in Premature Infants. Pediatrics.

[31] Nosarti C., Al-Asady M.H., Frangou S., Stewart A.L., Rifkin L., Murray R.M. (2002). Adolescents who were born very preterm have decreased brain volumes. Brain.

[32] Kesler S.R., Ment L.R., Vohr B., Pajot S.K., Schneider K.C., Katz K.H., Ebbitt T.B., Duncan C.C., Makuch R.W., Reiss A.L. (2004). Volumetric analysis of regional cerebral development in preterm children. Pediatr. Neurol..

[33] Peterson B.S., Vohr B., Staib L.H., Cannistraci C.J., Dolberg A., Schneider K.C., Katz K.H., Westerveld M., Sparrow S., Anderson A.W. (2000). Regional brain volume abnormalities and long-term cognitive outcome in preterm infants. JAMA.

[34] Maunu J., Parkkola R., Rikalainen H., Lehtonen L., Haataja L., Lapinleimu H., PIPARI Group (2009). Brain and ventricles in very low birth weight infants at term: A comparison among head circumference, ultrasound, and magnetic resonance imaging. Pediatrics.

